# Automated 3D Printing-Based Non-Sterile Compounding Technology for Pediatric Corticosteroid Dosage Forms in a Health System Pharmacy Setting

**DOI:** 10.3390/pharmaceutics17060762

**Published:** 2025-06-09

**Authors:** M. Brooke Bernhardt, Farnaz Shokraneh, Ludmila Hrizanovska, Julius Lahtinen, Cynthia A. Brasher, Niklas Sandler

**Affiliations:** 1 Department of Pharmacy and Pharmaceutical Sciences, St. Jude Children’s Research Hospital, Memphis, TN 38105, USA; brooke.bernhardt@stjude.org (M.B.B.); cindy.brasher@stjude.org (C.A.B.); 2 CurifyLabs Oy, Salmisaarenaukio 1, 00180 Helsinki, Finland; farnaz.shokraneh@curifylabs.com (F.S.); ludmila.hrizanovska@curifylabs.com (L.H.); julius.lahtinen@curifylabs.com (J.L.); 3 Pharmaceutical Sciences Laboratory, Åbo Akademi University, 20520 Turku, Finland

**Keywords:** compounding, automation, compounding system solution, pediatric glucocorticoid steroid therapy, personalized medicine, hydrocortisone, orodispersible films, tablets, hospital system pharmacy automation

## Abstract

**Background:** Pharmaceutical compounding remains a predominantly manual process with limited innovation, particularly in non-sterile applications. This study explores the implementation of an automated compounding platform based on 3D printing to enhance precision, efficiency, and adaptability in pediatric corticosteroid formulations. **Methods:** Personalized hydrocortisone dosage forms were prepared in a hospital pharmacy setting using a proprietary excipient base and standardized procedures, including automated dosing and syringe heating when required. Three dosage forms—3.2 mg gel tablets, 2.8 mg water-free troches, and 1.2 mg orodispersible films (ODFs)—were selected to demonstrate the platform’s versatility and to address pediatric needs for varying strengths and dosage types. All products were prepared using a reproducible semi-solid extrusion (SSE)-based workflow with the consistent API-excipient blending and automated deposition. **Results:** Analytical testing confirmed that all formulations met pharmacopeial criteria for mass and content uniformity. The ODF and troche forms achieved rapid drug release, exceeding 75% within 5 min, while the gel tablet showed a slower release profile, reaching 86% by 60 min. Additionally, in-process homogeneity testing across syringe printing cycles confirmed the consistent API distribution. **Conclusions**: The results support the feasibility of integrating automated compounding technologies into pharmacy workflows. Such systems can improve accuracy, minimize variability, and streamline the production of customized pediatric medications, particularly for drugs with poor palatability or narrow therapeutic windows. Overall, this study highlights the potential of automation to modernize non-sterile compounding, and to better support individualized therapy.

## 1. Introduction

Ensuring precise and individualized pediatric medication dosing remains a challenge in clinical practice [1,2]. The limitations of commercially available formulations often necessitate compounding to meet the unique needs of pediatric patients [3]. However, traditional manual compounding methods are prone to variability, are labor-intensive, and may not always ensure consistent dose accuracy [4,5]. To overcome these limitations, automated compounding systems can offer a viable solution for this challenge, offering enhanced accuracy, efficiency, standardization, and reproducibility in compounding pediatric drug preparations. Most advancements within the area of automated preparation and workflow automation have taken place in the field of sterile compounding during the past decades [6,7]. In sterile settings, automated compounding devices (ACDs) and robotic systems, such as gravimetric verification software, peristaltic pump-driven compounders, and robotic arms for cytotoxic drug preparation, have improved accuracy and reduced human error [8,9]. These systems provide integrated digital workflows, automated documentation, barcode verification, and real-time error detection—resulting in improved safety and reduced workload for pharmacy staff [10,11,12]. For example, the implementation of gravimetric compounding workflows has demonstrated substantial reductions in dose variability and compounding errors, with studies reporting error interception rates of 7.89% across hundreds of thousands of doses [13,14]. Researchers in European hospital settings have demonstrated that integrating prescribing, compounding, and administration software not only reduces transcription errors but streamlines interprofessional communication and increases output without adding to staff workload [12].

While sterile compounding automation is well-established in many hospital pharmacies, non-sterile compounding has lagged behind in automation. This domain remains heavily dependent on manual processes [15]. The slower adoption of automation in non-sterile compounding can be attributed to several persistent challenges, including the absence of standardized formulations, high variability in patient-specific preparations, and historically limited commercial incentives to invest in scalable technological solutions. However, recent innovations, such as automated 2D and 3D printing technologies, may be the key to bridge this gap. These digital manufacturing methods have shown the potential to improve drug content uniformity and enable precise dose customization, thus reducing the risk of human error and ensuring better adherence to pediatric dosing requirements [16]. In addition to improving quality control and dose consistency, digital drug manufacturing (e.g., 2D and 3D printing-based approaches) offer scalability and adaptability for hospital pharmacies [17]. Research on personalized pediatric pharmacotherapy suggests that flexible manufacturing platforms, such as automated printing and other digital compounding technologies, will play an increasingly important role in customized medication production [18]. Moreover, several studies have specifically demonstrated the advantages of 3D printing in pediatric drug delivery. For example, extrusion-based 3D printing has enabled the production of pediatric formulations with tailored shapes, sizes, and drug release profiles, improving palatability and compliance [19,20]. Clinical investigations have also shown that 3D-printed personalized medicines can be effectively used in children with rare metabolic disorders, highlighting their clinical relevance [21]. Technology’s capability to produce small batches or even single personalized doses makes it particularly suitable for hospital and pharmacy settings requiring on-demand pediatric medicines [22]. Furthermore, 3D printing allows for designing dosage forms with appealing shapes and colors, which can sometimes enhance the acceptability and adherence in pediatric patients [23].

This flexibility is particularly relevant for pediatric patients, where individualized dosing requirements necessitate adaptive drug formulation strategies. By leveraging automated dosing technologies, hospital pharmacies can ultimately enhance precision in pediatric drug dosing and achieve workflow efficiency, while maintaining regulatory compliance and patient safety. Examples of other automated or semi-automated or scalable compounding technologies e.g., small-scale tablet presses, semi-automatic capsule fillers, peristaltic pump-based liquid filling systems, and precision milling devices for powder-filled capsules or sachets also exist. These technologies can support increased efficiency and standardization for specific dosage forms but offer less flexibility in terms of dose customization, formulation versatility, and integration with digital compounding records when compared to 3D printing approaches. The integration of automated 3D and 2D printing technologies into pharmaceutical workflows demonstrates a shift toward more personalized, patient-centered medication solutions [16]. These technologies provide enhanced flexibility, reproducibility, and efficiency, enabling hospitals to produce tailored formulations on demand [6,17].

Orodispersible films (ODFs), gel tablets, and troches have gained traction as patient-friendly alternatives to conventional pediatric medications, such as solutions and suspensions, which can lead to dosing errors (e.g., at wards and by parents) [24]. ODFs, in particular, offer ease of administration, rapid dissolution, and accurate dosing for children with swallowing difficulties [25,26]. Each of these dosage forms can be reliably produced through automated 3D and 2D printing technologies.

The current study examined the application of a 3D printing-based automated CSS in developing pediatric dosage forms to be used within a real-world hospital setting. By integrating automation into pharmaceutical compounding, this study focuses on compounding gel tablets, water-free tablets (troches), and ODFs, addressing the relevant dosage forms for pediatric compounding. Hydrocortisone, a commonly prescribed corticosteroid in pediatric care, plays a vital role in managing conditions such as adrenal insufficiency, inflammatory disorders, and autoimmune diseases. Given its widespread use and the need for precise dose adjustments, ensuring consistent dose uniformity and bioavailability is essential to maintain therapeutic efficacy and prevent the risks associated with under- or overdosing. To validate the reliability and pharmaceutical quality of CSS-compounded formulations, this study incorporated in-process control (IPC) for mass uniformity, high-performance liquid chromatography (HPLC)-based chemical analysis, and in vitro dissolution testing following the USP methods of the compounded dosage forms. Ultimately, this research aims to advance the utilization of non-sterile personalized pediatric medicine through innovative 3D printing-based automated CSS, enabling hospital pharmacies to efficiently produce patient-specific medications with digital traceability.

## 2. Materials and Methods

**Figure 1:**
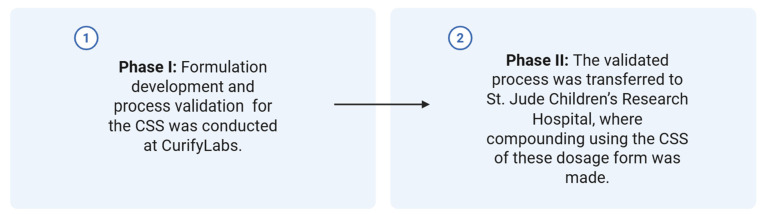
Two-phase implementation steps of the CSS technology in a health system pharmacy setting.

This study followed a two-phase approach to support the development, validation, and technology transfer of corticosteroid formulations produced using CSS technology (Figure 1).

**Phase I**—Product Development and Process Validation (CurifyLabs, Helsinki, Finland)

In this phase, the formulation and manufacturing methods were developed and validated. The process validation covered key manufacturing steps including the following:

- Mixing;
- Dosing;
- Packaging.

Additionally, comprehensive testing was conducted to ensure product quality and stability, including the following:

- Product stability studies to assess shelf-life and storage conditions, for up to six months, depending on the formulation, under room temperature conditions (approximately 20–25 °C, ambient humidity) to assess physical appearance and assay test;
- Dissolution testing to evaluate drug release characteristics;
- Syringe content homogeneity testing to ensure dose uniformity.

The detailed HPLC content uniformity and dissolution test methods are provided in Supplementary Materials S1 and S2, respectively.

**Phase II**—Technology Transfer and Final Compounding (St. Jude Children’s Research Hospital, Memphis, TN, USA)

After Phase I validation, the process was transferred to the receiving site. At St. Jude, the formulations were compounded according to the validated process. The finished dosage forms were then subjected to quality control testing to verify conformity to predefined specifications and to ensure product consistency post-transfer.

### 2.1. Materials

All materials used in the formulations of this study were of pharmaceutical grade including the excipient bases.

**Phase I:** The excipient bases (CurifyLabs, Helsinki, Finland) used for the automated dosing process were formulated with hydrocortisone (sourced from Caesar & Loretz GmbH, Hilden, Germany) along with polysorbate 80 (Caesar & Loretz GmbH, Hilden, Germany). Three distinct pharmaceutical excipient bases under the CuraBlend^®^ brand were utilized in the formulation’s development, all provided by CurifyLabs, Helsinki, Finland (Figure 1).

The CuraBlend^®^ gel tablet base is primarily composed of purified water, gelatin, and cocoa butter, providing a soft, chewable matrix suitable for immediate-release and pediatric formulations.

The CuraBlend^®^ water-free tablet/troche excipient base consisted mainly of polyethylene glycol, cocoa butter, and maltodextrin, offering a moisture-free base ideal for enhancing the stability of hygroscopic or hydrolytically sensitive drugs.

The CuraBlend^®^ ODF excipient base featured polyethylene oxide, sorbitol, and maltodextrin as its key components, forming a fast-dissolving, flexible film suitable for orodispersible dosage forms.

**Phase II:** St. Jude Children’s Research Hospital formulation active pharmaceutical ingredient (API) of hydrocortisone and polysorbate 80 were sourced from Medisca (Montreal, QC, Canada). HPLC-grade acetonitrile and methanol were obtained from Fisher Scientific (Loughborough, UK). All chemicals and reagents used in this study were of analytical grade to ensure precision and reliability in the analysis (Figure 1).

For blister packaging, 3/16” Mini Medi-Cup^®^ Plus™ Blisters (MD425, The MediDose Group, PA, USA) were selected for the blistering of gel tablets and ODF films. The blister lids were LaserLabel™ “25” Lid-Label^®^ Cover Sheets from MediDose. Additionally, Gako Blister & Lid (100 × 15) from Gako International GmbH, Scheßlitz Germany, were utilized for the packaging of water-free tablets, ensuring safe and efficient storage. Sterilized single-use PVC syringes (100 mL) with a Luer-lock mechanism were used (CurifyLabs, Helsinki, Finland) in the printing step.

### 2.2. Methods

#### 2.2.1. Compounding System Solution (CSS) Technology

CurifyLabs’ Compounding System Solution (CSS, CurifyLabs, Helsinki, Finland) is a fully integrated and modular platform for non-sterile pharmaceutical compounding by combining pharmaceutical excipient bases, a software platform with order handling and a formulation library, advanced robotics with 3D printing and planetary mixing, packaging material and integrated quality control. This system enables the automated production of personalized medications with precise dosing and form flexibility, while ensuring traceability, digital batch records, and real-time process monitoring for safety and compliance. As a result, CSS allows pharmacies and hospitals to produce quality controlled, personalized dosage forms, such as gummies, troches, tablets, liquids, films, rectal and vaginal suppositories, and capsules—all in a decentralized, efficient, standardized, and controlled environment.

**Figure 2:**
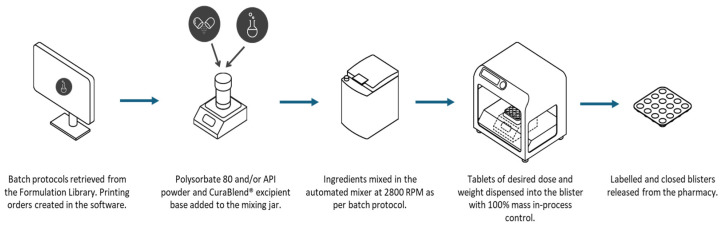
Overview of the non-sterile compounding workflow with the Compounding System Solution.

The CSS is designed to support both clinical workflow requirements and regulatory expectations, including those outlined in the USP <795> for non-sterile compounding. It enables standardized formulation handling, precise dosing, and consistent mixing, which can help streamline compounding procedures and reduce variability in practice. The system also facilitates compliance with documentation and quality assurance standards by incorporating digital batch records, access controls, and tools for assigning Beyond-Use Dates (BUDs) in accordance with the USP <795> criteria. By providing structured, traceable processes, CSS contributes to safer, more consistent compounding within clinical environments. Figure 2 shows the typical workflow of the CSS system.

#### 2.2.2. Mixing of the APIs with the Excipient Bases

In Phase I all formulations were prepared as follows:

Hydrocortisone gel tablet formulations were prepared by weighing 2.0% (*w*/*w*) hydrocortisone and 2.0% (*w*/*w*) polysorbate 80, followed by the addition of 96.0% (*w*/*w*) gel tablet excipient base into a mixing container. Each blend was homogenized using an automated planetary mixer (PM 140, Gako Deutschland GmbH, Schesslitz, Germany) operated at 2800 rpm for 10 min. Following mixing, the melted formulations were transferred into sterilized single-use 100 mL PVC syringes equipped with a Luer-lock mechanism (CurifyLabs, Helsinki, Finland).

Troche formulations containing hydrocortisone were prepared by weighing 0.75% (*w*/*w*) hydrocortisone and 99.25% (*w*/*w*) troche excipient base. Each formulation was placed in a water bath maintained at 60–70 °C for 5 min to maintain a molten state. The mixtures were then homogenized using the PM 140 planetary mixer at 2800 rpm for 2 min, followed by a second 5-min interval in the 60–70 °C water bath. The melted troche formulations were subsequently transferred into sterilized single-use 100 mL PVC syringes with a Luer-lock mechanism (CurifyLabs, Helsinki, Finland).

Orally disintegrating film (ODF) formulations were prepared by weighing 0.30% (*w*/*w*) hydrocortisone and adding 99.70% (*w*/*w*) of the ODF CuraBlend^®^ base into a mixing container. The API-excipient mixtures were mixed in the PM 140 planetary mixer at 2800 rpm for 2 min. Final formulations were then transferred into sterilized single-use 100 mL PVC syringes fitted with a Luer-lock mechanism (CurifyLabs, Helsinki, Finland).

The 100 mL syringe acts as the reservoir for the formulation and the dispensing/printing of the final dosage form.

#### 2.2.3. Automated Preparation of the Dosage Forms

The compounding of the dosage forms (Phase I) and tech transfer compounding (Phase II) were carried out using the CSS and the Pharma Printer (CurifyLabs Oy, Helsinki, Finland), an extrusion-based 3D Printer device equipped with a dispensing head optimized for semi-solid extrusion. Specific dose options for each formulation are created within the dedicated software prior to compounding during the batch record design process to be stored within the formulation library. Parameters for temperature, material extrusion, and density were optimized for each formulation. Moderate printing temperatures of 41, 25, and 60 °C were used for the gel tablets, ODFs, and troches, respectively. When ready to compound, the system’s dedicated software allows users to flexibly select the desired tablet/film weight from the formulation library [27].

#### 2.2.4. Syringe Homogeneity Study

A 100 g batch was prepared in Phase I and loaded into a 100 mL syringe of a 3D Pharma Printer in phase I. The printing process was carried out at a controlled temperature (40 °C), continuously dispensing unit doses with predefined target weights. To assess uniformity within the syringe during the printing process, samples were collected at three key stages:

Start of Printing: Three units were collected immediately after the process began, with one unit selected from each of the first, third, and last rows of the blister pack.

Midpoint of Printing: Three additional units were collected following the same cross-sectional sampling pattern.

End of Printing: Four units were collected—three from the first, third, and last rows, and one randomly selected from any position.

**Figure 3:**
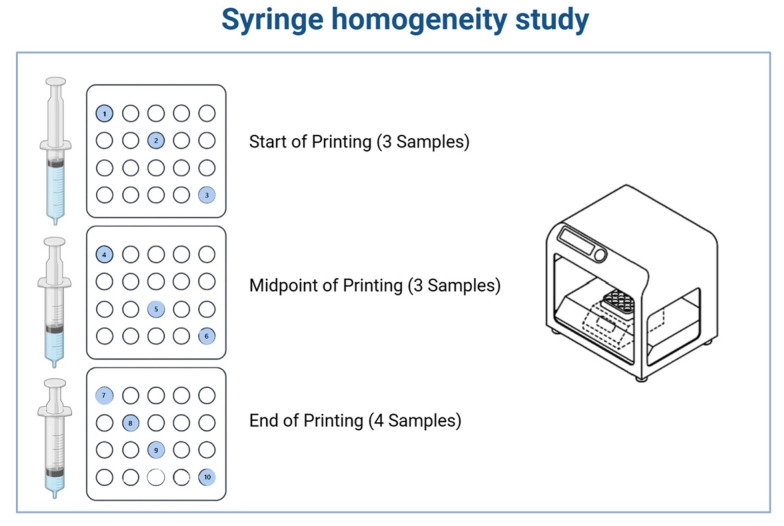
Syringe homogeneity study and sampling plan for evaluating content uniformity during semi-solid 3D printing. A 100 g batch was prepared and loaded into a 100 mL syringe of a 3D Pharma Printer. The printing process was carried out at a controlled temperature, continuously producing unit doses. The colored and numbered circles indicate the samples that were used for the syringe homogeneity test.

This sampling strategy was designed to evaluate consistency in content throughout the printing process and to ensure the homogeneity of the material dispensed from the syringe (Figure 3).

For the syringe homogeneity testing across different base formulations, the following compositions were selected:

- 0.75% hydrocortisone in a water-free troche base;
- 2% hydrocortisone in CuraBlend^®^ gel base;
- 0.3% hydrocortisone in an ODF base.

All formulations were printed into 400 mg dosage units and subsequently tested for content uniformity to evaluate syringe-to-syringe homogeneity.

#### 2.2.5. Phase II: Technology and Process Transfer

**Figure 4:**
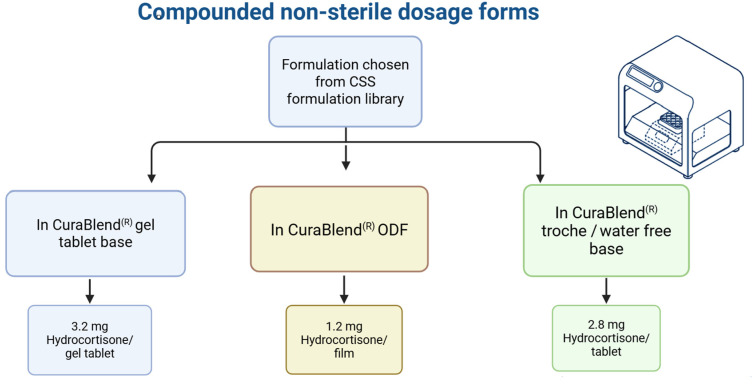
Overview of printed pharmaceutical samples produced during technology transfer, categorized by excipient base type and dosage form in Phase II.

In Phase II, technology and process transfers were carried out using pre-defined formulations from the CSS formulation library. As visualized in Figure 4, the transfer included three CuraBlend^®^ base types: gel tablet base, water-free base, and ODF base. Each blend was successfully printed on-site using extrusion-based semi-solid 3D printing, demonstrating compatibility with the target equipment and verifying process reproducibility across various compositions and formats.

Following production, all printed samples were submitted to CurifyLabs’ laboratory for content uniformity and dissolution testing, and for confirmation about product quality and in vitro performance.

#### 2.2.6. Dosing Accuracy and Mass Variation for Tablets and Films

Dosing accuracy measures the percentage of tablets and films within a batch that conform to the specified mass variation criteria. It is calculated by determining the proportion of units that meet the mass variation limits relative to the total number of units, and expressing the result as a percentage as follows:

Dosing Accuracy = (the number of tablets/films within specification/total number of tablets/films) × 100

The mass variation specifications are derived from the mass uniformity test criteria outlined in the European Pharmacopoeia (Ph. Eur.) 2.9.5. According to these guidelines, all tablets and films undergo mass uniformity evaluation, with the acceptance criteria set as follows:

- 7.5% (m/m) for 200 mg or less tablets or films;
- 5.0% (m/m) for 300, 400, and 500 mg tablets or films.

#### 2.2.7. Assay, Content Uniformity, and Dissolution Testing

Hydrocortisone content and dissolution profiles were analyzed using a validated HPLC method and standard USP procedures. A Waters AQUITY ARC HPLC system (Waters, Milford, MA, USA) equipped with a BEH C18 column was used. For assay and content uniformity, samples were analyzed at 240 nm, while dissolution testing was performed at 230 nm using USP Apparatus 2 (paddle method) at 50 rpm and 37 °C. The dissolution medium used was water, with volumes of 500 mL for tablets and 900 mL for ODF films, and time points ranged from 0 to 60 min depending on the dosage form.

Test solutions for content uniformity were prepared to target 100–200 ppm, with acceptance values (AV) calculated per the Ph. Eur. 2.9.40 and the USP <905> guidelines. Full method parameters, gradient conditions, and dissolution settings are detailed in Supplementary Materials S1 and S2. For analytical testing, 6 tablets were used for dissolution, 10 tablets for content uniformity (as per Ph. Eur. 2.9.40/USP <905>), and 18 tablets for assay evaluation. All tests were conducted on a single GMP batch, with each test performed once unless otherwise specified.

#### 2.2.8. Data Analysis

For all quality control evaluations, descriptive statistical analysis was performed using Microsoft Excel. Mean values and standard deviations (SD) were calculated for each dosage form, as follows:

- Mass uniformity was assessed using 25 units per blend, with acceptance based on the Ph. Eur. 2.9.5 and the USP <905> limits: ±7.5% for units ≤200 mg and ±5.0% for units between 300 and 500 mg.
- Content uniformity was evaluated using 10 units, and AV was calculated according to the USP <905> guidelines. The AV formula is as follows:

AV = |M − X| + ks

where:

- M = X, if 98.5 ≤ X ≤ 101.5;
- M = 98.5, if X < 98.5;
- M = 101.5, if X > 101.5;
- K = 2.4;
- S = standard deviation.

A result is acceptable if AV ≤ 15.

- Dissolution testing was conducted using 6 units per dosage form, in accordance with USP Apparatus 2, and results were expressed as mean ± SD. The acceptance criterion was Q ≥ 75% drug release within 30 min.
- Homogeneity assessments were conducted during the syringe printing process at the beginning, mid-point, and end, using triplicate samples per time point. Consistency was evaluated based on mean, SD, and AV at each stage.

## 3. Results

Three dosage forms with various doses were chosen from the CSS formulation library for preparation and analysis. The quality control results are presented in the sections below.

### 3.1. Mass Uniformity

**Figure 5:**
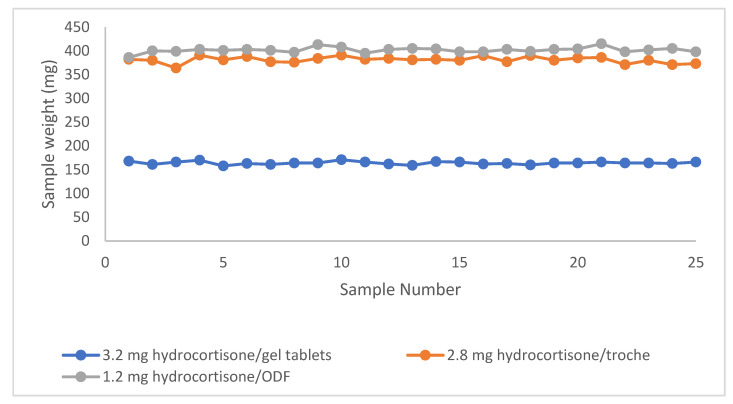
Individual in-process weights (n = 25) of printed dosage units for each blend (gel tablets, troches, ODFs). The data illustrated compliance with pharmacopeial mass uniformity criteria based on weight variation thresholds.

Mass uniformity of the three printed pharmaceutical blends was evaluated in phase II according to the acceptance criteria set by both the European Pharmacopoeia (Ph. Eur.) 2.9.5 and the USP <905> Uniformity of Dosage Units. These standards require dosage units to fall within ±7.5% of the target weight for formulations ≤200 mg, and within ±5.0% for those between 300 and 500 mg. As shown in Figure 5, individual unit weights (n = 25 per blend) are plotted for each dosage form, demonstrating consistent mass distribution within the pharmacopeial limits. This expected weight includes the required amount of the API and the respective base. The expected weight is pre-determined when the product is created in the CSS formulation library. We analyzed 25 units for each dosage form—3.2 mg hydrocortisone gel tablets, 2.8 mg hydrocortisone troches, and 1.2 mg hydrocortisone ODFs—to assess the weight-based uniformity as a proxy for precise dosing. The average sample weights were 164.1 mg for the gel tablets, 381.0 mg for the troches, and 401.6 mg for the ODFs. Standard deviations of the dosage form weights were within acceptable limits, with values of 3.1 mg, 6.7 mg, and 5.7 mg, respectively (n = 25), indicating consistent mass distribution across units. All blends achieved 100% dosing accuracy relative to target fill weights, as visualized in Figure 5.

### 3.2. Content Uniformity Test by HPLC

HPLC testing confirmed content uniformity across all three dosage forms. All AV were below the USP <905> threshold of 15, indicating compliance with pharmacopeial criteria. The observed AV values were 10 for ODFs, 8 for gel tablets, and 11 for troches. As shown in Table 1, all blends met the criteria for content uniformity, with the API recovery ranging from 105% to 109%, with standard deviations below 2%. The table presents the final blend weights, the mean API content percentages, the associated standard deviations, and the calculated AV values for each dosage form.

### 3.3. Dissolution Test

**Figure 6:**
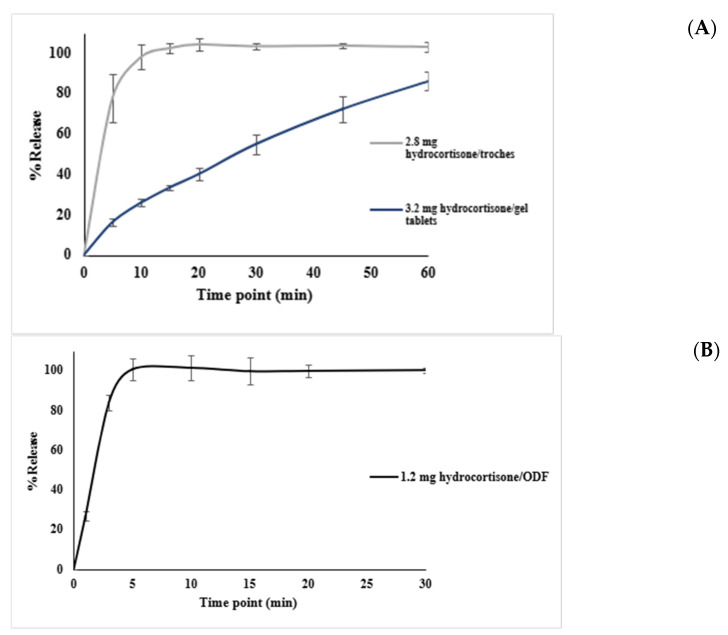
Dissolution profiles of printed formulations containing hydrocortisone in various CuraBlend^®^ base types and dosage forms: (**A**) dissolution of 2.8 mg hydrocortisone troche prepared using CuraBlend^®^ water-free troche base and dissolution of 3.2 mg hydrocortisone gel tablets prepared with CuraBlend^®^ gel tablet base; (**B**) dissolution of 1.2 mg hydrocortisone ODF formulated with CuraBlend^®^ ODF base. The vertical lines indicate standard deviations in each time point.

Dissolution testing in phase II was conducted to evaluate the drug release profiles of the hydrocortisone formulations prepared using different excipient bases. The objective was to confirm compliance with the USP dissolution criteria, which required that at least 75% of the drug (Q ≥ 75%) be released within 30 min. As shown in Figure 6, dissolution profiles for all three dosage forms—troche, gel tablet, and ODF—demonstrated distinct release patterns based on the base type used. Each formulation achieved ≥75% drug release within 30 min, meeting the USP specifications. The data are presented as mean ± SD from three replicates per dosage form.

All samples demonstrated formulation-dependent release profiles, with data expressed as mean ± SD (n = 3).

Table 2 summarizes the full dissolution profiles of the hydrocortisone formulations. The 2.8 mg troche released over 77% of the drug within 5 min, and exceeded 100% by 15 min. The 1.2 mg ODF rapidly achieved complete release, reaching over 83% within 3 min, and over 100% by 5 min. By contrast, the 3.2 mg gel tablet displayed a slower release pattern, reaching only 55% at 30 min, and 86% by 60 min. These profiles reflect formulation-dependent release behavior, with all products meeting or exceeding the USP criterion of Q ≥ 75% drug release within 30 min where applicable.

### 3.4. Syringe Homogeneity Test

The API content was consistent across beginning, mid-point, and end of printing for all dosage forms. The 0.75% troche excipient blend maintained tight clustering (AV = 1, n = 10), the 2% gel tablet excipient blend showed minor mid-point variability (AV = 6, n = 10), and the 0.3% film excipient blend showed higher initial variability but stabilized later (AV = 5, n = 10). As summarized in Table 3, the homogeneity of the API distribution during the syringe printing process was evaluated using replicate samples taken at three time points. The table presents the raw API content values, the calculated average ± standard deviation, and the AV, providing a detailed view of blend consistency throughout the print cycle.

## 4. Discussion

**Figure 7:**
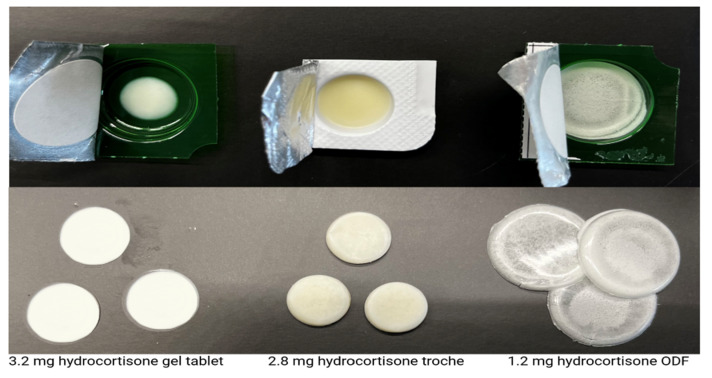
Visual appearance of 3D-printed hydrocortisone dosage forms using different excipient bases in a blister cavity (above), and separately on a flat surface (below). The diameters of the gel tablets, troches, and the ODFs are 8 mm, 13 mm, 30 mm, respectively, and their thickness is in the same order approximately 3 mm, 4 mm, and 200 microns, respectively.

The reliability of the CSS 3D printing platform in producing pediatric hydrocortisone formulations is supported by detailed quality control results. Mass uniformity testing demonstrated precise weight control across 25 units of each dosage form, all falling within pharmacopeial specifications (Ph. Eur. and USP), with standard deviations of 3.1 mg (gel tablets), 6.7 mg (troches), and 5.7 mg (ODFs). An HPLC-based content uniformity analysis yielded AV between 8 and 11 for all formulations, confirming compliance with the USP <905>. Dissolution studies further reinforced the consistency of the system. The orodispersible film (ODF) showed rapid release, reaching over 100% of drug content by 5 min; the troche met the USP Q ≥ 75% threshold within 5 min, and exceeded 100% by 15 min; while the gel tablet did not reach 75% release within 30 min (only 55% at 30 min), it achieved 86.1% by 60 min. Given the intended use of the gel tablet as a chewable dosage form, the slower in vitro release is acceptable, as chewing would enhance drug liberation in vivo. Figure 7 shows the visual appearance of the different dosage forms.

All formulations demonstrated 100% dosing accuracy relative to their target fill weights. These results confirm the reliability and precision of the automated medication production system in producing uniform dosage units across diverse formulation types. The consistency observed across all three products—spanning various concentrations and dosage forms—highlights the precision and reproducibility of the printing method. Figure 5 visually reinforces this uniformity, supporting the suitability of the CSS and 3D printing for pharmaceutical compounding. These findings align with the prior studies on digital manufacturing, which have demonstrated high reproducibility in mass uniformity for personalized dosage forms [28,29]. The ability to maintain such consistency across diverse formulations also suggests that 3D printing could address challenges in traditional manufacturing, such as batch-to-batch variability [24]. In general, as reported by Sandler Topelius et al. [27] and Shokraneh et al. [15], the approach taken with CSS technology allows for 100% quality control of the weight of the finished product, which is not a component of traditional compounding practices in compounding pharmacies making patient tailored medicines, significantly improving the safety of the compounding procedure.

Ensuring consistent drug content across different pediatric dosage forms is critical for maintaining therapeutic efficacy and patient safety. This study, in Phase II, evaluated the content uniformity of hydrocortisone formulations prepared using the studied excipient bases across various dosage forms, including gel tablets, water-free tablets, and ODFs. The analysis focused on different final tablet weights (160 mg to 400 mg) and formulation bases to determine the impact of compounding variables on dose uniformity.

The results demonstrated that most formulations met AV < 15, confirming their suitability for pediatric administration. The 1.2 mg hydrocortisone ODF and 3.2 mg hydrocortisone gel tablets exhibited excellent content uniformity, maintaining low standard deviation (SD) and relative standard deviation (RSD%) values (Table 1). These findings are supported by several studies demonstrating that hydrophilic bases and excipients enhance drug solubility and uniformity compared to anhydrous or less hydrophilic systems. For example, hydrophilic hypromellose-based composites and hydrophilic nanofibers have been shown to significantly improve the dissolution rates and uniformity of poorly water-soluble drugs, largely due to their amorphous nature and compatibility with drug molecules, which promote rapid and uniform drug release [30,31]. Additionally, the use of hydrophilic polymers, such as poloxamers and hydroxypropyl-β-cyclodextrin, has been found to increase the solubility, stability, and release rate of drugs, with the hydrophilic environment facilitating better drug incorporation and distribution compared to more anhydrous or hydrophobic systems [31,32]. These results collectively indicate that hydrophilic bases are advantageous for enhancing both solubility and uniformity in drug delivery formulations [33].

The differences in dosage forms and excipient bases appeared to influence the variability observed across the batches. Water-free formulations, which do not rely on aqueous solvents, posed challenges in achieving uniform drug dispersion, particularly in higher-dose, larger tablets (400 mg). By contrast, the gel-based and ODF formulations provided more stable drug distribution, as evidenced by their low AV and RSD% values.

Among all the tested dosage forms, the 1.2 mg hydrocortisone CuraBlend^®^ ODF exhibited the fastest dissolution profile, achieving 75% drug release within 3 min and reaching complete release by 10 min. This rapid dissolution is attributed to the high surface area and thin structure of the orodispersible film, which promotes quick hydration and disintegration, making it ideal for immediate-release applications. These results align with previous research highlighting the suitability of ODFs for rapid drug delivery, particularly in pediatric populations [34,35].

The 2.8 mg hydrocortisone troche tablet met the USP dissolution criteria, releasing at least 75% of the drug within 30 min, and showing complete release by 45 min. The dissolution rate was slower than that of the ODF format, which may be explained by the anhydrous nature of the formulation and the slightly larger mass, requiring more time to absorb moisture and disintegrate.

By contrast, the hydrocortisone 3.2 mg gel tablets did not meet the USP criteria for conventional tablets, with 55% released at 30 min. Complete release was achieved by 75 min, indicating that higher drug loading in a smaller dosage unit may hinder disintegration and delay dissolution. Additionally, the gel-forming nature of the base may contribute to slower erosion and drug release rates. It is important to note that the gel tablets are intended to be chewable, which introduces a limitation in interpreting dissolution test results, as chewing significantly accelerates disintegration and drug release in vivo. Therefore, in vitro dissolution may underestimate actual release performance for these forms. This observation is supported by studies suggesting that chewable formulations require modified dissolution testing to account for mechanical breakdown [36].

In summary, physical characteristics and base composition play a role in dissolution behavior, although all dosage forms show immediate release behavior as follows:

- ODF: thin, water-soluble, fast disintegrating;
- Water-Free troche: slightly slower, but also efficient due to the high solubility of the base;
- Gel Tablets: form a gel upon hydration, which can slow the release moderately unless chewed.

It is important to note that these formulations represent concentrations that can be used to produce the typical oral therapeutic doses of hydrocortisone used at St Jude Children’s Research Hospital. The formulations were also designed for developmental and stress-testing purposes, including the evaluation of release characteristics at higher drug loads and under challenging formulation conditions.

Comparatively, the 1.2 mg hydrocortisone ODF exhibited the most rapid dissolution, reaching over 100% drug release within 5 min, which was attributed to its thin film structure and hydrophilic base. The 2.8 mg troche followed closely, achieving over 75% release by 5 min and complete dissolution by 15 min, demonstrating strong performance despite its larger mass. By contrast, the 3.2 mg gel tablet showed the slowest release (55% at 30 min), though still acceptable for a chewable formulation where in vivo disintegration is expected to be faster. Regarding content uniformity, all formulations were within pharmacopeial limits (AV < 15), with the gel tablet showing the lowest AV (8), suggesting the most consistent drug distribution, followed by ODF (AV = 10) and troche (AV = 11). Mass uniformity results also supported high reproducibility, with gel tablets having the lowest standard deviation in weight. These findings demonstrate formulation-dependent performance, influenced by both matrix composition and structural characteristics.

We conducted the syringe homogeneity test in phase I to evaluate the API distribution consistency across various corticosteroid formulations during the automated dosing process. Samples were collected at the beginning, mid-point, and end of printing to identify potential variability.

Table 2 provides a detailed comparison of the API content across three critical time points during the 3D printing process—beginning, mid-point, and end—for three different semi-solid formulations: 0.75% hydrocortisone troche, 2% hydrocortisone gel tablet, and 0.3% hydrocortisone film. This test was conducted to evaluate within-syringe homogeneity during continuous printing under controlled thermal and flow conditions.

- **The 0.75% Hydrocortisone Troche Blend**

The API content remained highly consistent across all printing stages. The average content values were 101.0% (±0.3%) at the beginning, 101.4% (±0.3%) at the mid-point, and 101.5% (±0.4%) at the end. The calculated AV was one, indicating excellent uniformity and blend stability during the syringe-based printing process.

- **The 0.3% Hydrocortisone Film Blend**

The film formulation showed the highest variability at the start of the printing process (99.3% ± 3.6%), likely due to the low API concentration and the thin, delicate matrix, which may be more sensitive to early-stage inconsistencies in extrusion. However, uniformity improved notably at the mid-point (101.3% ± 0.8%) and at the end (101.5% ± 0.7%) of printing, suggesting stabilization of the system as thermal and mechanical equilibrium was reached. The overall AV of five remained within the acceptable limits, though the initial fluctuation highlights the potential benefit of implementing a pre-conditioning or priming step to minimize dose variability in the first units printed.

- **The 2% Hydrocortisone Gel Tablet Blend**

The gel formulation exhibited acceptable content uniformity throughout the printing process. The API levels were consistent at the beginning (103.2% ± 1.3%) and the end (103.7% ± 0.4%) of printing, while a slightly higher variability was observed at the midpoint (104.2% ± 2.7%). Despite this increased fluctuation, all values fell within the acceptable pharmacopeial limits. The calculated AV of six was the highest among the three formulations, yet still indicative of acceptable homogeneity.

In the gel tablet formulation, a higher standard deviation (2.7%) was observed at the mid-point. This may be related to the rheological behavior of the gel base, where temporary inhomogeneity or slight drug redistribution can occur during sustained extrusion.

All tested hydrocortisone blends maintained acceptable content uniformity during the automated dosing process. This test was performed with particular attention to the troche formulations, which are considered more susceptible to sedimentation due to their semi-solid, non-gelled consistency. By contrast, gel-based formulations possess intrinsic viscosity and gelling properties that naturally minimize particle settling and reduce the risk of inhomogeneity during printing. Although minor variations were observed in some formulations, these were formulation-dependent and did not compromise the overall uniformity or quality of the printed dosage forms. Collectively, these findings support the reproducibility and robustness of the automated dosing system in delivering consistent, patient-specific medications.

In contrast to earlier demonstrations of pharmaceutical 3D printing focused on adult-use applications and simple capsule filling, which have evaluated the feasibility of automated capsule compounding, our study advances the field by delivering a validated, integrated solution tailored specifically for pediatric pharmacotherapy. While prior research has established proof-of-concept for 3D printing in hospital pharmacies [21,37], our work goes further by rigorously assessing multiple pediatric-appropriate dosage forms—including chewable gel tablets, orodispersible films (ODFs), and troches—under realistic use-case scenarios.

Our findings demonstrate that the CSS 3D printing platform ensures high reproducibility, dosing precision, and formulation versatility across a variety of base materials and drug concentrations, all supported by pharmacopeial-quality control metrics (USP <905>, <711>). In particular, our evaluation of the in-syringe API distribution over the entire dosing cycle addresses a critical gap in the literature by confirming blend stability during thermal extrusion, a process limitation rarely studied in pediatric formulations [19]. Furthermore, this work incorporates non-destructive in-process monitoring and pre-validated formulation libraries—features that align with regulatory expectations for pharmaceutical process analytical technology (PAT) and good manufacturing practice (GMP) adaptation [22,38].

Importantly, the formulation-dependent variability observed in dissolution behavior across different excipient matrices provides new insight into excipient selection strategies for pediatric dose individualization. Our results complement the earlier dissolution modeling studies for orodispersible films and semi-solids [39] but extend their applicability to real-world personalized dosing scenarios in clinical pharmacy.

Taken together, this work offers tangible technical and clinical advancements for integrating 3D printing into pediatric compounding practice. It not only demonstrates the feasibility of on-demand, patient-specific manufacturing but outlines a standardized, automated pathway that minimizes human error and supports safer, reproducible treatment personalization. These capabilities are especially crucial for vulnerable populations, like children, where flexible dose titration, flavor masking, and accurate delivery are critical to therapy success [40,41,42].

These collective findings highlight the effectiveness of the CSS 3D printing system in producing precise and uniform hydrocortisone formulations tailored for pediatric use. Despite minor blend-specific variations, all tested dosage forms demonstrated acceptable quality attributes in terms of mass uniformity, content consistency, and printing homogeneity. The system’s ability to handle different excipient bases and drug concentrations supports its practical applicability in personalized medicine. This study advances the field of pediatric 3D-printed formulations by demonstrating the practical application of an automated compounding platform capable of producing age-appropriate, personalized dosage forms with high precision and reproducibility. Compared to traditional manual compounding methods, the CSS standardizes preparation steps, minimizes human error, and enables rapid, on-demand manufacturing using pre-validated formulation libraries. These capabilities are particularly valuable in pediatric care, where accurate dosing, flexible formulation design, and improved palatability are critical. A video in the Supplementary Materials comparing the CSS platform to manual capsule filling further illustrates the operational benefits and efficiency gains achieved through automation. Collectively, this work supports the integration of digital compounding technologies into routine pharmacy practice, aligning with broader goals in precision medicine and pharmaceutical quality assurance.

## 5. Conclusions

This study demonstrates the feasibility, reliability, and versatility of using automated compounding technologies based on 3D printing in combination with excipient bases with optimal properties for producing pediatric-specific dosage forms. Through the systematic evaluation of key pharmaceutical quality attributes, including mass uniformity, content uniformity, dissolution performance, and syringe homogeneity, the results indicated these dosage forms could be consistently prepared using the 3D Pharma Printer for accurate and precise dosing.

Mass uniformity assessments confirmed that all tested formulations met the acceptance criteria outlined in the European Pharmacopoeia and the USP <905>. All three dosage forms—gel tablets (3.2 mg), troches (2.8 mg), and ODFs (1.2 mg)—demonstrated excellent mass uniformity across 25 samples. The average weights were 164.1 mg, 381.0 mg, and 401.6 mg, respectively, with low standard deviations (3.1–6.7 mg), indicating consistent production. Accuracy was 100% for all formulations, confirming reliable dose delivery. These doses were produced in Phase II, in the hospital setting, and further demonstrate that the CSS has accuracy that can be reproduced in different settings.

Content uniformity, as determined by HPLC, further supported the reliability of the system. All tested formulations exhibited an AV below the critical threshold of 15, confirming their suitability for pediatric administration.

These results present the robustness of the CuraBlend^®^ excipients—particularly gel and ODF bases—in supporting uniform API distribution, even at low or high drug concentrations. By contrast, larger water-free formulations tended to exhibit slightly higher variability, underlining the influence of formulation base and tablet size on dose uniformity.

Dissolution testing revealed clear distinctions in drug release behavior across different dosage formats. The ODF formulation achieved >75% drug release within just 3 min, with complete release by 10 min, reflecting rapid disintegration and high surface area. The troche formulation, while still compliant with the USP criteria, showed slower profiles depending on formulation characteristics. The gel tablet formulation did not meet the USP criteria for dissolution. It released only 55% of the drug by 30 min, though it reached full release by 75 min—suggesting that higher drug loading in smaller units and gel-forming matrices may delay disintegration and dissolution. The impact of chewing a gel tablet on the dissolution rate, as would happen in vivo, was not demonstrated by this test.

Syringe homogeneity tests, conducted across beginning, mid-point, and end of the printing process, confirmed the consistent API distribution. The AV values ranged from one to six, with formulations such as the 3 mg hydrocortisone troche exhibiting exceptionally low variability (AV = one), even in semi-solid formats typically prone to sedimentation. These results reinforce the precision of the automated compounding system solution and its suitability for reproducible manufacturing and for taking pharmacy compounding to the next level in terms of quality and precision.

This study contributes to the advancement of automated 3D printing based pharmaceutical compounding in pediatric care by evaluating the feasibility of CSS technology for the standardized production of hydrocortisone formulations. The research emphasizes the importance and potential of automation, quality control, and patient-centered medication design. By addressing potential improvement areas in hospital-based pediatric medication preparation processes and workflows for non-sterile compounding, this study supports the broader implementation of flexible, automated compounding solutions in clinical pharmacy practice.

## Figures and Tables

**Table 1:** Content uniformity results.

Product	Printed Weight (mg)	Average Content (%), n = 10	SD (%)	AV	Meets Criteria
1.2 mg hydrocortisone ODF	400	107.0	1.7	10	Yes
2.8 mg hydrocortisone troche	373	108.6	1.4	11	Yes
3.2 mg hydrocortisone gel tablet	160	105.2	1.9	8	Yes

**Table 2:** Dissolution profiles of hydrocortisone dosage Forms.

Time (min)	2.8 mg Troche (%) ± SD, n = 6	3.2 mg Gel Tablet (%) ± SD, n = 6	1.2 mg ODF (%) ± SD, n = 6
0	0 ± 0	0 ± 0	0 ± 0
1	—	—	26.8 ± 5.1
3	—	—	83.9 ± 8.7
5	77.8 ± 11.7	16.3 ± 1.8	100.6 ± 5.6
10	98.1 ± 6.2	26.0 ± 1.8	101.4 ± 3.0
15	102.5 ± 2.3	33.6 ± 1.2	99.7 ± 2.5
20	104.3 ± 3.2	40.1 ± 3.2	99.8 ± 3.1
30	103.5 ± 1.7	55.0 ± 4.9	100.2 ± 2.9
45	103.8 ± 1.2	72.3 ± 6.7	—
60	103.1 ± 2.4	86.1 ± 4.6	—

**Table 3:** Content uniformity results of syringe homogeneity test.

Product Name	Printing Stage	API Content (%)	Average (%) ± SD	Acceptance Value
**0.75% Hydrocortisone troche blend**	Beginning of Printing	101.0%, 101.3%, 100.8%	101.0% ± 0.3% (n = 3)	1
	Mid-Point of Printing	101.7%, 101.2%, 101.5%	101.4% ± 0.3% (n = 3)	
	End of Printing	101.4%, 101.0%, 102.0%, 101.7%	101.5% ± 0.4% (n = 4)	
**2% Hydrocortisone gel tablet blend**	Beginning of Printing	103.0%, 102.0%, 104.7%	103.2% ± 1.3% (n = 3)	6
	Mid-Point of Printing	101.7%, 107.0%, 103.7%	104.2% ± 2.7% (n = 3)	
	End of Printing	103.4%, 104.0%, 104.0%, 103.3%	103.7% ± 0.4% (n = 4)	
**0.3% Hydrocortisone film blend**	Beginning of Printing	101.1%, 101.7%, 95.1%	99.3% ± 3.6% (n = 3)	5
	Mid-Point of Printing	100.6%, 101.3%, 102.2%	101.3% ± 0.8% (n = 3)	
	End of Printing	102.1%, 102.2%, 101.2%, 100.6%	101.5% ± 0.7% (n = 4)	

## Data Availability

The data supporting the findings of this study are available from the corresponding author or CurifyLabs upon reasonable request.

## Supplementary Materials

The following supporting information can be downloaded at: https://www.mdpi.com/article/10.3390/pharmaceutics17060762/s1, Table S1: HPLC Method Parameters for Hydrocortisone Assay; Table S2: Dissolution Test Conditions for Hydrocortisone Dosage Forms. Video S1: Video of demonstrating time savings of capsule vs. CSS technology.

## Abbreviations

The following abbreviations are used in this manuscript:

API	Active Pharmaceutical Ingredient
CSS	Compounding System Solution
HPLC	High-Performance Liquid Chromatography
IPC	In-Process Control
ODF	Orodispersible Film
Ph. Eur.	European Pharmacopoeia
RSD%	Relative Standard Deviation (Percentage)
SD	Standard Deviation
USP	United States Pharmacopeia
