# Supplementary material for: Automated 3D Printing-Based Non-Sterile Compounding Technology for Pediatric Corticosteroid Dosage Forms in a Health System Pharmacy Setting
Source: Pharmaceutics. 2025 Jun 9;17(6):762. doi: 10.3390/pharmaceutics17060762 (PMC12197057; doi:10.3390/pharmaceutics17060762)
Supplement: Supplementary file 1 [file pharmaceutics-17-00762-s001.zip › pharmaceutics-3660739-supplementary.pdf]

pharmaceutics

S1 (Appendix A)

A.1. Assay and Content Uniformity Method

A Waters AQUITY ARC High-Performance Liquid Chromatography (HPLC) system (Massachusetts, USA) equipped with a Quaternary Solvent Manager-R (ACQ-rQSM), degasser, autosampler (ACQ-rFTN), and photodiode-array detector (2998 PDA) was used for analysis. Data acquisition and processing were performed using Empower Workstation software. The detailed HPLC analytical method is provided in the Supplementary Information (Table S1).

A.2. Sample and Standard Preparation for Content Uniformity Testing

Based on the printed sample strengths, each formulation was processed to prepare test solutions targeting a final concentration of 100–200 ppm for content uniformity analysis. Standard stock solutions (500 ppm) were prepared by dissolving 25 mg of API in 50 mL MilliQ water, followed by sonication and vortexing to ensure complete dissolution and homogeneity. Working standards (50–100 ppm) were obtained by diluting 1–2 mL of stock solution in a 10 mL volumetric flask. Tablet samples were dissolved in appropriately sized volumetric flasks (25–100 mL) based on their weight (200–500 mg), using a water bath at 50°C. After cooling and vortexing, all samples were filtered through 0.25 µm syringe filters and transferred to HPLC vials for analysis.

The Acceptance Value (AV) was determined following the guidelines outlined in Chapter 2.9.40 "Uniformity of Dosage Units" of the European Pharmacopoeia (Ph. Eur.) and USP <905>. This calculation, based on the mean content limits specified in the relevant monograph, represents the arithmetic mean of uniformity results across individual dosage units.

The AV is computed using a formula that incorporates the acceptability constant (coverage factor) and the standard deviation (S) from the analysis of 10 units. To meet the acceptance criteria, AV must not exceed 15. The formula used is:

$$AV = |M - X| + ks$$

$$M = X, \text{ if } 98.5 \leq X \leq 101.5$$

$$M = 98.5, \text{ if } X < 98.5$$

$$M = 101.5, \text{ if } X > 101.5$$

K=2.4

S=standard deviation

Table S1. HPLC Method Parameters for Hydrocortisone Assay.

Parameter	Hydrocortisone Assay
Column	BEH C18 (2.5 µm, 100 mm × 4.6 mm, Waters, UK)
Mobile Phase	Milli-Q Water: Methanol (50:50, v/v)
Flow Rate	0.7 mL/min
Injection Volume	2 µL
Detection Wavelength	240 nm
Column Temperature	35°C

S2 (Appendix B)

B.1. Dissolution Method

The dissolution analysis was carried out on a multi-bath (n = 6) dissolution test apparatus 2, 50 rpm for speed (paddle) with dissolution tester DT 128 (Erweka GmbH, Germany) in accordance with the United States Pharmacopoeia (USP) general methods. All measurements were carried out using a Waters AQUITY ARC HPLC system equipped with a Quaternary solvent manager-R (ACQ-rQSM), a degasser, autosampler (ACQ-rFTN), and a photodiode-array detector (2998 PDA). The detector was set at 230 nm. Withdrawn samples underwent filtration through disposable cellulose syringe filters (0.2 μm) to obtain clear solutions. The sinking conditions were maintained by replacing fresh media after each withdrawal from each vessel. Samples were analyzed by HPLC following the method described in Supplementary Table S2.

Table S2. Dissolution Test Conditions for Hydrocortisone Dosage Forms

Parameter	Hydrocortisone Tablets	Hydrocortisone ODF Films
Apparatus	USP Apparatus 2 (Paddle)	USP Apparatus 2 (Paddle)
Dissolution Medium	Water	Water
Medium Volume	500 mL	900 mL
Rotation Speed	50 rpm	50 rpm
Temperature	37 °C	37 °C
Sampling Time Points (minutes)	0, 1, 3, 5, 10, 15, 20, 30	0, 5, 10, 15, 20, 30, 45, 60

Video S1: Video Demonstrating Time Savings

Link: https://www.youtube.com/watch?v=OIYzFfdTlAw
